# Physicochemical and Biological Properties of Oxovanadium(IV), Cobalt(II) and Nickel(II) Complexes with Oxydiacetate Anions

**DOI:** 10.1007/s12011-014-0170-x

**Published:** 2014-12-07

**Authors:** Dariusz Wyrzykowski, Anna Kloska, Joanna Pranczk, Aneta Szczepańska, Aleksandra Tesmar, Dagmara Jacewicz, Bogusław Pilarski, Lech Chmurzyński

**Affiliations:** 1 Faculty of Chemistry, University of Gdańsk, Wita Stwosza 63, 80-308 Gdańsk, Poland; 2 Faculty of Biology, University of Gdańsk, Wita Stwosza 59, 80-308 Gdańsk, Poland; 3 Cerko Sp. z o.o. Sp. K, Al. Zwycięstwa 96/98, 81-451 Gdynia, Poland

**Keywords:** Oxydiacetate complexes, Cytoprotective activity, Antimicrobial activity, Potentiometric titration, Conductometric titration, Stability constant

## Abstract

The potentiometric and conductometric titration methods have been used to characterize the stability of series of VO(IV)-, Co(II)- and Ni(II)-oxydiacetato complexes in DMSO-water solutions containing 0–50 % (*v*/*v*) DMSO. The influence of DMSO as a co-solvent on the stability of the complexes as well as the oxydiacetic acid was evaluated. Furthermore, the reactivity of the complexes towards superoxide free radicals was assessed by employing the nitro blue tetrazolium (NBT) assay. The biological properties of the complexes were investigated in relation to their cytoprotective activity against the oxidative damage generated exogenously by using hydrogen peroxide in the Human Dermal Fibroblasts adult (HDFa) cell line as well as to their antimicrobial activity against the bacteria (*Bacillus subtilis*, *Escherichia coli*, *Enterococcus faecalis*, *Pseudomonas aeruginosa*, *Staphylococcus aureus*, *Staphylococcus epidermidis*). The relationship between physicochemical and biological properties of the complexes was discussed.

## Introduction

Polycarboxylate metal complexes have attracted the interest of many research teams because of their extensive applications in wide areas from material to biological sciences [1–7]. The flexible nature of the ligands permits designing and syntheses of new coordination compounds of different architecture and topologies [8]. Moreover, the crystal engineering of coordination-polymeric solids enables a topological control over the crystalline form and hence allows to obtain the material of desired electronic, magnetic, optical or catalytic properties for solid state technologies [9, 10].

The oxydiacetate ion belongs to dicarboxylate ligands, and moreover, it contains the ethereal donor atom that is capable to take part in the coordination to the central atom. The flexibility of this ligand enables to adopt, depending on the kind of metal ion and a method of synthesis, different geometrical conformations from a planar (*mer* coordination) to folded about the M-O vector (*fac* coordination) [11, 12]. However, ab initio calculations reveal that the mer ↔ fac energetic barrier is small enough and thus the co-existence of both isomers in the solution at the equilibrium is possible [13]. The flexibility of the geometric transformations around the metal centre as well as the ability of metal ion to undergo the one-electron reduction by superoxide followed by the reoxidation of the reduced metal ion by a second superoxide ion are considered to be one of the factors responsible for the superoxide dismutase (SOD) mimetic activity of the complexes. For instance, oxydiacetate copper complexes, [Cu(ODA)(bipy)(H_2_O)]∙4H_2_O and [Cu(ODA)(4-pic)(H_2_O)]∙2H_2_O where bipy and 4-pic denote 2,2′-bipyridine and 4-picoline, respectively, exhibit a considerable SOD activity [14, 15]. Furthermore, several metal complexes comprising oxydiacetate as a primary ligand and α-diimine as an auxiliary ligand, [M(ODA)(bipy)(H_2_O)]∙xH_2_O [*M* = Cu^2+^ or Ni^2+^, *x* = 4; Cr^2+^, *x* = 3; Co^2+^, *x* = 2] and [VO(ODA)(bipy)]∙2H_2_O as well as [M(ODA)(4-pic)(H_2_O)]∙xH_2_O [*M* = Cu^2+^ or Ni^2+^, *x* = 2; Cr^2+^, *x* = 3; Co^2+^, *x* = 4] and [Fe(ODA)(4-pic)]Cl, have been found to exhibit antibacterial activities [14, 15].

The subject of our continuous interest are polycarboxylate metal complexes since it has been found that the oxydiacetate complexes of VO(IV), Co(II) and Ni(II) can protect effectively the mouse hippocampal neuronal cell line (HT22) against an oxidative stress induced by H_2_O_2_ [16]. Hydrogen peroxide is the most frequently mentioned agent responsible for the oxidative stress in neurodegenerative diseases, brain strokes and atherosclerotic diseases. The present study constitutes a continuation of our earlier efforts on the characterization of physicochemical and biological properties of the oxydiacetate complexes [16–18]. The potential application of low-molecular weight coordination compounds as therapeutic superoxide dismutase (SOD) mimics for the scavenging of superoxide free radicals (O_2_^−^) during the treatment of diseases of different aetiology and for the protection of healthy tissues was the reason that prompted us to embark on these studies.

In the present work, we report a study on cytoprotective properties of [VO(ODA)(H_2_O)_2_], [Co(ODA)(H_2_O)_2_]·H_2_O and [Ni(ODA)(H_2_O)_3_]·1.5H_2_O against the oxidative damage generated exogenously in the Human Dermal Fibroblasts adult (HDFa) cell line as well as their antimicrobial activities against the bacteria (*Bacillus subtilis*, *Escherichia coli*, *Enterococcus faecalis*, *Pseudomonas aeruginosa*, *Staphylococcus aureus*, *Staphylococcus epidermidis*). Furthermore, to get a better insight into physicochemical and biological properties of the complexes, their stability in water and DMSO-water binary systems were investigated. To the best of our knowledge, there are no reports on stabilities of these complexes in the solutions of different physicochemical properties. The obtained results may be helpful for the in-depth understanding of the relationship between physicochemical and biological properties of the complexes under study.

## Material and Methods

### Syntheses of the Complexes

All reagents used for the syntheses were of analytical grade and were used without further purification. They were as follows: VO(acac)_2_ (≥98 %), CoCl_2_·6H_2_O (≥98 %), NiCl_2_·6H_2_O (≥98 %) and 2,2′-oxydiacetic acid (H_2_ODA) (≥98 %). The syntheses of [VO(ODA)(H_2_O)_2_], [Co(ODA)(H_2_O)]∙H_2_O and [Ni(ODA)(H_2_O)_3_]∙1.5H_2_O were carried out according to the procedures described in the literature [13, 19]. The compositions of the compounds were established on the basis of the elemental analysis of carbon and hydrogen (Vario EL analyzer Cube CHNS). Anal. Calcd for [VO(ODA)(H_2_O)_2_] C, 20,44 %, H, 3,44 %: found: C, 20,42 %, H, 3,45 %; [Ni(ODA)(H_2_O)_3_]∙1.5H_2_O: C, 17.65 %, H, 4.83 %: found: C, 17.45 %, H, 4.79 %; [Co(ODA)(H_2_O)_2_]·H_2_O C, 19,60 %, H, 4.12 %; found: C, 19,48 %, H, 4,10 %.

### Potentiometric Titrations (PT)

Potentiometric titrations were performed in a 30-mL thermostated (298.15 ± 0.10 K) cell using the Cerko Lab System microtitration unit fitted with a 5-mL Hamilton’s syringe, a pH combined electrode (Schott–BlueLine 16 pH type) and a self-made measuring cell equipped with a magnetic stirrer. The temperature was controlled using the Lauda E100 circulation thermostat. In aqueous solutions, the electrode was calibrated according to IUPAC recommendations [20]. In binary systems (10–50 % *v/v* DMSO:H_2_O), the electrode calibration was performed by the Gran’s method [21] using the GLEE program [22]. For this purpose, the HClO_4_ solution was titrated with tetrabutylammonium hydroxide (Bu_4_NOH) solution both at the same mole fraction of organic solvent (10–50 (*v*/*v*%) DMSO). The syringe was calibrated by a weight method. The double distilled water of conductivity approximately 0.18 μS/cm was used throughout for the preparation of aqueous solutions. Other reagents used in the experiments were purchased from Sigma-Aldrich. They were as follows: VOSO_4_ (≥99.99 % trace metals basis), Co(NO_3_)_2_·6H_2_O (ACS reagent, ≥99 %), Ni(NO_3_)_2_·6H_2_O (99.999 %) and 2,2′-oxydiacetic acid (H_2_ODA, 98 %). All the solutions were prepared immediately before measurements. The compositions of the titrand solutions used in the experiments were as follows: (a) 2 mM Ni^2+^ (Co^2+^ or VO^2+^), 5 mM H_2_ODA, 5 mM HClO_4_, (b) 2.5 mM H_2_ODA (for binary systems). The solutions (V_o_ = 5.0 mL) were potentiometrically titrated with the standardized 0.098 M NaOH solution or with the standardized 0.025 M Bu_4_NOH solution (in binary systems) in the pH range from 2.5 to 11.0. The titrant was added to the titrand in increments of 0.01 mL, with a pause of 60 s. Each titration was repeated at least three times in order to check the reproducibility of the data. The stability constants of the complexes were determined using the Hyperquad2008 program [23]. The concentration distribution of various complex species existing in the solution as a function of pH was obtained using the HySS program [24].

### Conductometric Titrations (CT)

Conductometric measurements were accomplished on the Cerko Lab System microtitration unit fitted with a 5-mL Hamilton’s syringe, a CD-201 conductometric cell with the constant *k* = 0.096 cm^−1^ (HYDROMET) and a self-made measuring cell equipped with a magnetic stirrer. The conductometric electrode was standardized with conductivity standards (aqueous KCl solutions) of conductivity 84 and 200 μS/cm, purchased from Hamilton Company. The measurements were carried out at 298.15 ± 0.10 K controlled by the Lauda E100 circulation thermostat. The titrant (5 mM [Co(ODA)(H_2_O)] or [Ni(ODA)(H_2_O)_3_]) was added to the titrand (0–50 % *v/v* DMSO-H_2_O) both at the same mole fraction of organic solvent, in increments of 0.01 mL, with a pause of 60 s. Each titration was repeated at least three times in order to check the reproducibility of the data. The limiting molar conductance values (Λ_o_) and the association constants (log*K*_ass_) for the nickel(II) and cobalt(II) complexes were computed using the Shedlovsky’s [25] and Pitt’s [26, 27] method. The physicochemical parameters of the binary solvents used in the calculations were taken from the literature [28–33]. They are collected in Table 1.

**Table 1:** The relative permittivity *ε*, density *σ* and viscosity *η* for DMSO-water mixtures at 298.15 K

DMSO (*v/v*%)	*ε*	*σ* [g∙cm^−3^]	*η* (mPa∙s)
1	78.36	0.997	0.89
10	77.75	1.011	1.07
20	76.96	1.026	1.38
30	76.21	1.042	1.79
40	75.45	1.057	2.35
50	74.70	1.072	3.00

### The Scavenging of Superoxide Radicals (NBT Method)

Nitro blue tetrazolium (NBT, 98 % purity), KO_2_ (96 % purity) and 18-Crown-6 (99 % purity) were purchased from Sigma-Aldrich. The superoxide scavenging activity of the complexes was determined by using their ability to inhibit the reduction of nitro blue tetrazolium (NBT) by superoxide ions. UV–vis measurements were carried out by using the Perkin-Elmer Lambda 650 spectrophotometer equipped with the temperature control—Peltier system with a scan accuracy of 1 and 1-nm slit width. The measurements were carried out in DMSO solution at 288.15 K according to the method described in the literature [34]. Superoxide free radicals were generated by dissolving 6.5-mg portion of KO_2_ powder into DMSO (50 mL) together with 90 mg of 18-crown-6-ether. NBT (10 mg) was dissolved directly in DMSO (10 mL). In general, 1.5 mL of DMSO/KO_2_/18-crown-6 mixture was added to 0.5 mL of the sample to be assayed. The mixture was kept for 20 min and then 0.1 mL of NBT solution was added while stirring. The absorbance was monitored spectrophotometrically at 560 nm. The control test was carried out under the same experimental condition under assumption that KO_2_ and 18-crown-6 were absent in DMSO.

### The Cell Line and Culture Conditions

The Human Dermal Fibroblasts adult (HDFa) cell line (Cascade Biologics) was used in all experiments. Unless otherwise specified, cells were grown in the Dulbecco’s modified Eagle’s medium (DMEM) supplemented with 10 % fetal bovine serum (FBS) and 1× antibiotic and antimycotic solutions (all purchased from Sigma) and incubated at 310.15 K in the humidified 5 % CO_2_ atmosphere.

### The Cytotoxicity Assay

HDFa cells were plated at 6 × 10^3^ cells per well in a 96-well tissue culture plate and incubated overnight at 310.15 K to allow the attachment. The growth medium was substituted with the medium supplemented with tested complex compounds. After 24- or 48-h incubation, the cell viability was assessed using the MTT solution (3-(4,5-dimethylthiazol-2-yl)-2,5-diphenyltetrazolium bromide [Sigma]) at a concentration of 1 mg/mL prepared in the RPMI-1640 medium (Sigma). After 2-h incubation at 310.15 K, the amount of purple formazan product, dissolved in dimethylsulfoxide (DMSO) (Sigma), was quantified by measuring the absorbance at 550 nm. The cell viability was calculated as relative to cultures incubated with the medium only (non-treated control), and the LC_25_, LC_50_ and LC_75_ (lethal concentration for 25, 50 and 75 % of cells, respectively) index values were determined from dose–response curves using four parameter logistic equations.

### The Proliferation Assay

The antiproliferative activity of compounds was assessed using the Cell Proliferation ELISA, BrdU (colorimetric) kit (Roche Applied Science). Cells were seeded at a number of 10^3^ cells per well of 96-well tissue culture plate and incubated overnight at 310.15 K. The growth medium was substituted with the medium supplemented with tested compounds, and after 5 days of incubation, labelling with 5-bromo-2′-deoxyuridine (BrdU) was performed for another 24 h. Following steps were performed according to the manufacturer’s protocol. The cell proliferation was calculated as relative to cultures incubated with medium only (non-treated control), and the IC_25_, IC_50_ and IC_75_ (concentrations causing, respectively, 25, 50 and 75 % inhibition of cell proliferation) index values were determined from dose–response curves using four parameter logistic equations.

### The Determination of Cytoprotective Activity Against the Oxidative Damage

Cells were plated at 2 × 10^3^ cells per well of 96-well tissue culture plate. After an overnight incubation, cells were washed with DMEM without FBS and next treated with 1 mM hydrogen peroxide (H_2_O_2_) (Sigma) together with different concentrations of tested complex compounds or with 1 mM H_2_O_2_ alone to induce the oxidative damage to cells resulting in the necrotic death. Mixtures were prepared in the growth medium (DMEM) without FBS. Cells treated with the growth medium only were used as a control of normal viability. After 1-h incubation at 310.15 K, the medium was removed and cells were washed with the growth medium containing 10 % FBS to stop the H_2_O_2_ activity. Next, either the cell viability was assessed using the MTT solution (see previous paragraph) or additional incubation of cells in DMEM containing 10 % FBS for another 48 h was performed and then cells were tested for the viability (the MTT assay). The cytoprotective activity of complex compounds against the oxidative damage was calculated relatively to untreated control cells, not exposed to H_2_O_2_.

### Bacterial Strains and Culture Media

The antimicrobial activity of complex compounds was assessed against *Bacillus subtilis, Escherichia coli, Enterococcus faecalis*, *Pseudomonas aeruginosa*, *Staphylococcus aureus*, *Staphylococcus epidermidis* (all strains were from the collection of the Department of Molecular Biology, University of Gdańsk). All bacteria were cultured in the Luria-Bertani (LB) broth at 310.15 K in the air with shaking. LB agar plates were prepared by the supplementation of LB broth with the 1.5 % bacteriological agar. Stock solutions of complex compounds were freshly prepared in the LB broth at the concentration of 25 mg/mL.

### The Antimicrobial Activity of the Complexes

The antimicrobial activity was assessed with the broth dilution method. The LB broth supplemented with the twofold diluted amount of tested compounds (dilution range 1–2048 μg/mL) was inoculated with 10^6^ cfu of bacteria cells per well of 96-well plate. Cultures were incubated at 310.15 K with shaking for 18–20 h in the air, and the bacterial growth was estimated by measuring the optical density at 600 nm with the LB broth as a background control. A minimum inhibitory concentration (MIC) was defined as the lowest concentration of the tested compound (in μg/mL) at which there was no visible growth of bacteria compared to the growth observed for the inoculated LB broth without any supplementation. To estimate a minimum bactericidal concentration (MBC), 0.1 mL of bacterial cultures exhibiting no visible growth were spread on LB agar plates and after an overnight incubation at 310.15 K, the number of colonies was counted. The concentration (in μg/mL) at which no colonies were observed was defined as MBC.

### The Disc Diffusion Test

Aliquots of 0.05 mL of bacterial culture at the optical density at 600 nm of 0.1 (inoculum 5 × 10^6^ cells per plate) were spread on LB agar plates. Sterile filter paper discs (of 6 mm diameter) were impregnated with solutions of complex compounds prepared in the deionized water at the following amounts: 0.1, 0.2, 0.5 and 1.0 mg per disc and placed on agar plates. After an overnight incubation at 310.15 K, zones of bacterial growth inhibition were measured. Neomycin at the amount of 0.1 mg/disc was used as a control.

## Results and Discussion

### The Stability of the Complexes in Water and DMSO-Water Solutions

**Fig. 1:**
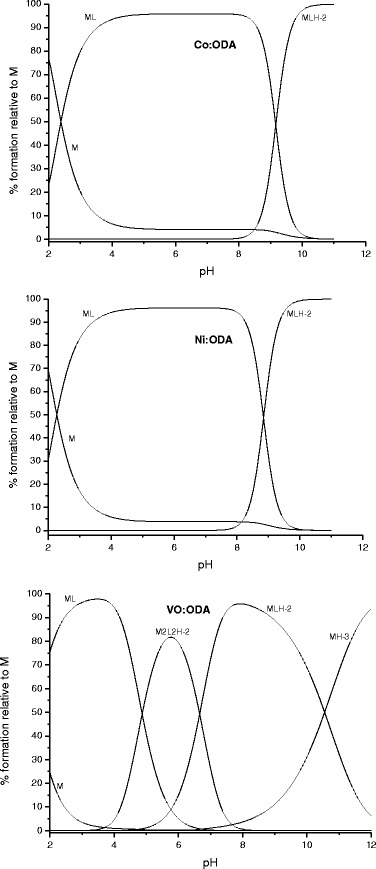
Concentration distribution curves of the complexes as a function of pH (M:L = 1:1) calculated based on the stability constants listed in Table 2

The equilibrium constants defined by Eqs. (1) and (2):

1 $$ \mathrm{p}\mathrm{M}+\mathrm{q}\mathrm{L}+\mathrm{r}\mathrm{H}={\mathrm{M}}_{\mathrm{p}}{\mathrm{L}}_{\mathrm{q}}{\mathrm{H}}_{\mathrm{r}} $$

2 $$ {\mathit{\mathsf{\beta}}}_{\mathrm{p}\mathrm{qr}}=\frac{\left[{\mathrm{M}}_{\mathrm{p}}{\mathrm{L}}_{\mathrm{q}}{\mathrm{H}}_{\mathrm{r}}\right]}{{\left[\mathrm{M}\right]}^{\mathrm{p}}{\left[\mathrm{L}\right]}^{\mathrm{q}}{\left[\mathrm{H}\right]}^{\mathrm{r}}} $$

(where M is VO^2+^, Co^2+^ or Ni^2+^, L denotes the oxydiacetate ion, H is the proton and p, q, r are stoichiometric coefficients for the reaction) were refined by least-squares calculations using the Hyperquad2008 (version 5.2.19) computer program [20]. For the oxovanadium(IV) system, the formation of the hydroxo complexes of VO(IV) were taken into account in the calculations of the stability constants. The following formation constants were taken from the literature [35] and were fixed: log*β*_10–1_ = −5.94, log*β*_20–2_ = −6.95 and log*β*_10–3_ = −18. The equilibrium models presented in Table 2 have given the best fitting of the calculated data to the experimental ones. Species distributions as a function of pH (molar ratio M:L equals 1:1) are shown in Fig. 1.

**Table 2:** Logarithms of the stability constants (log*β*_pqrs_) of binary complexes of VO(IV), Co(II) and Ni(II) with oxydiacetate ion (ODA) obtained by adapting the equilibrium model to PT data

No.	Species	log*β*_pqr_	M = Co^2+^	M = Ni^2+^	M = VO^2+^
1	LH_2_	log*β*_012_	7.26 (±0.06)	6.97 (±0.01)	7.25 (±0.05)
2	LH	log*β*_011_	4.16 (±0.09)	4.12 (±0.01)	4.20 (±0.05)
3	ML	log*β*_110_	3.45 (±0.11)	3.52 (±0.02)	5.08 (±0.04)
4	MLH_−2_	log*β*_11–2_	−14.88 (±0.14)	−14.17 (±0.06)	−6.45 (±0.04)
5	M_2_L_2_H_−2_	log*β*_22–2_	–	–	1.65 (±0.15)

The standard deviations are given in parenthesis

It has been found that the stability constants of the cobalt(II) and nickel(II) complexes are equal in the range of experimental error but for the oxovanadium(VI) complex, the stability constant is ca. 1 order higher (Table 2). This finding is in agreement with the hard and soft acids and bases (HSAB) theory whereby the oxovanadium(IV) ion as a hard acid forms more stable (thermodynamically) complexes with ligands containing donor atoms of a high electronegativity (such as oxygen atoms in the oxydiacetate ion). Conversely, cobalt(II) and nickel(II) as borderline acids form with the oxydiacetate ions less stable complexes than VO(IV). However, due to the hydroxide competition with the oxydiacetate ion for VO(IV), the coordination centre easily undergoes a hydrolysis and the resulting hydroxo complex species are formed (Table 2). Thus, in contrary to the Co(II) and Ni(II) complexes, VO(ODA) exists in the narrow range of pH and above pH of 5.5 hydroxo species predominate (Fig. 1).

The stability of the complexes under study was tested in the DMSO-water solution containing 0-50 % (*v*/*v*) DMSO. The investigation of solvent effects on the stability of the complexes enables to predict their behaviour in the systems of different than pure water physicochemical properties. Besides, the DMSO-water mixture is of a special interest among scientists not only because of its manifold applications as the solvent and reaction media in biology and medicine but also because of its important role in biochemical processes [36–38].

**Fig. 2:**
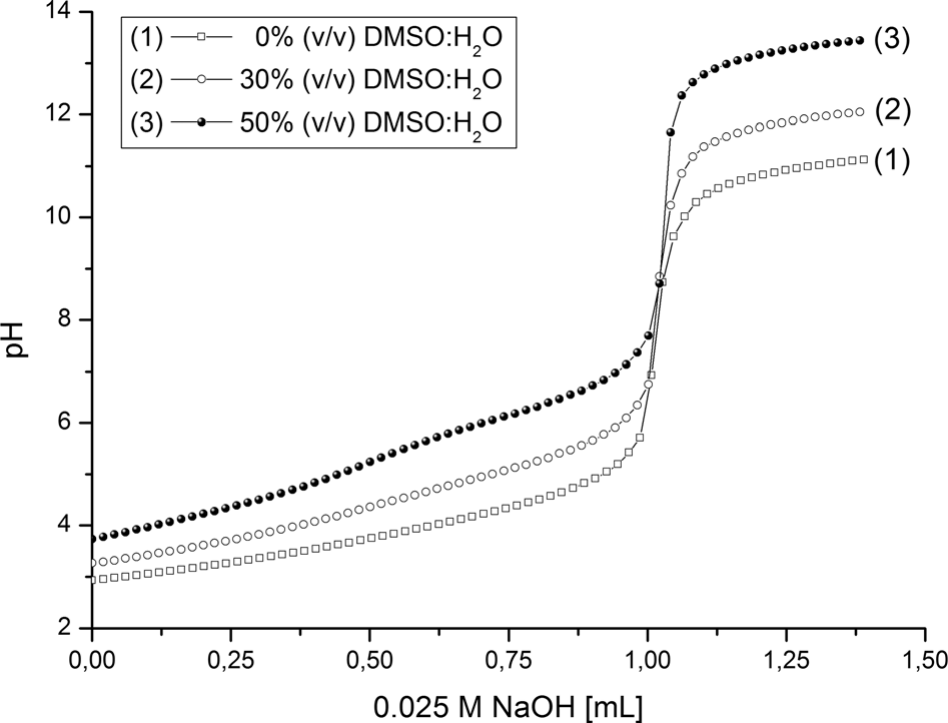
pH-metric titration curves of H_2_ODA at 298.15 K in **a** water, **b** DMSO-water 30 % (*v/v*), **c** DMSO-water 50 % (*v/v*) binary system

To assess the influence of DMSO as a co-solvent on the stability of the complexes, its effect on the dissociation constants of H_2_ODA was investigated potentiometrically in the first instance. The representative pH-titration curves for oxydiacetic acid at 298.15 K in 0, 30 and 50 % (*v*/*v*) DMSO-water binary systems are shown in Fig. 2, whereas the calculated dissociation constants are collected in Table 3. In the calculations, the ionization constants of the solvents, p*K*_s_, were taken from the literature [39].

**Table 3:** The dissociation constants of oxydiacetate acid (expressed as p*K*_a1_ and p*K*_a2_) of different DMSO-water mixtures at 298.15 K

DMSO(% *v/v*)	p*K*_a1_ (H_2_ODA = HODA^−^ + H^+^)	p*K*_a2_ (HODA^−^ = ODA^2−^ + H^+^)	p*K*_s_
0	2.90 (±0.05)	4.28 (±0.08)	14.0
10	3.11 (±0.04)	4.52 (±0.02)	14.09
20	3.28 (±0.02)	4.69 (±0.02)	14.26
30	3.42 (±0.04)	4.91 (±0.02)	14.40
40	3.61 (±0.03)	5.16 (±0.02)	14.66
50	3.76 (±0.03)	5.46 (±0.03)	14.82

The standard deviations are given in parenthesis

As shown in Table 3, the p*K*_a_’s values of H_2_ODA increase in proportion to the increase of DMSO component in the system. Thus, the protonated species (H_2_ODA and HODA^−^) are becoming more stable (less acidic) as a dielectric constant of a reaction medium is getting lower along with the increase of DMSO content. Generally, it is observed that the stability of the metal complexes increases with the increasing basicity of the ligand [40]. For this reason, the formation of more stable complexes may be expected along with the enrichment of medium in the DMSO component. This assumption has subsequently been verified by studying association constants of the complexes determined from conductometric measurements in DMSO-water mixtures containing 0-50 % (*v*/*v*) DMSO. Unfortunately, for the oxovanadium(IV) complex reproducible, reliable data could not been obtained in the binary DMSO-water system due to the too low conductivity of the VO(ODA) solutions. In the presence of DMSO, the VO(ODA) complex becomes even more stable than in water and the concentration of species due to its dissociation as well as the concentration of the resulting hydroxo complex are too low to conduct conductometric measurements.

The limiting molar conductance values (Λ_o_) and the association constants (log*K*_ass_) for the nickel(II) and cobalt(II) complexes were computed using Shedlovsky’s [25] and Pitt’s [26, 27] equations and are consequently collected in Table 4.

**Table 4:** The limiting molar conductance (Λ_o_) values in S cm^2^ mol^−1^ and the association constants (log*K*_ass_) of the oxydiacetate cobalt(II) and nickel(II) complexes in DMSO-water mixtures at 298.15 K

Method	Parameter	DMSO-water (% *v/v*)					
		0	10	20	30	40	50
NiODA							
Shedlovsky	Λ_o_	216.10(±0.74)	161.00(±0.95)	119.83(±0.83)	90.63(±1.37)	51.85(±0.61)	28.80(±0.48)
	*logK*_ass_	3.33 (±0.01)	3.47 (±0.01)	3.59 (±0.01)	3.74 (±0.01)	4.05 (±0.02)	4.15 (±0.03)
Pitts	Λ_o_	216.74(±1.08)	161.75(±0.93)	114.72(±1.55)	91.35(±1.33)	52.00(±0.50)	29.49(±0.92)
	*logK*_ass_	3.34 (±0.01)	3.48 (±0.01)	3.56 (±0.01)	3.75 (±0.01)	4.05 (±0.01)	4.17 (±0.04)
CoODA							
Shedlovsky	Λ_o_	223.49(±1.45)	171.55(±0.74)	133.47(±1.00)	92.16(±1.07)	55.24(±0.84)	28.52(±1.58)
	*logK*_ass_	3.35 (±0.01)	3.49 (±0.01)	3.77 (±0.01)	3.89 (±0.01)	4.09 (±0.03)	4.22 (±0.02)
Pitts	Λ_o_	226.58(±1.32)	173.97(±0.60)	134.29(±1.09)	92.53(±0.96)	57.02(±0.71)	29.74(±1.46)
	*logK*_ass_	3.38 (±0.01)	3.51 (±0.01)	3.77 (±0.01)	3.89 (±0.01)	4.05 (±0.01)	4.32 (±0.03)

The conductometric data have shown that both limiting molar conductance (Λ_o_) values and the association constants change approximately linearly with the increase of the amount of DMSO in the system. The dissociation of the complexes takes place to a lesser extent. It manifests itself both in the decrease of molar conductance and in the increase of the association constants. Thus, the neutral complexes of the type M(ODA) are more stable in media with low dielectric constants (the higher content of DMSO in the system the lower dielectric constant of the medium). At the same time, the solvation of the ionic species by the solvent is becoming less likely and the metal-ligand interactions predominate.

### The Reactivity of the Complexes Towards Superoxide Free Radicals

The ability of the complexes to scavenge superoxide anion radicals was evaluated by the NBT assay. The measurements were carried out in the DMSO solution in which KO_2_ was dissolved via the use of a crown ether to generate superoxide radicals (see Material and Methods).

**Fig. 3:**
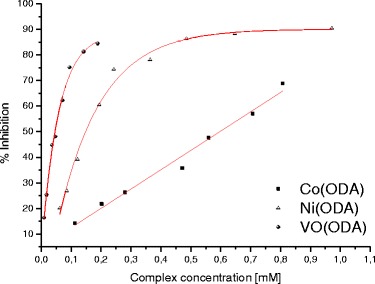
The superoxide free radicals scavenging activity of the complexes

The plots of the percentage of inhibition versus complex concentrations are presented in Fig. 3. It has been found that all the complexes exhibit the activity towards scavenging of superoxide anions. The results obtained from the NBT assay are in agreement with those obtained earlier by using the CV method [16]. Both methods have proved that the complexes are capable to scavenge superoxide free radicals. A linear relation was obtained for the cobalt(II) complex (Fig. 3). For the oxovanadium(IV) and nickel(II) complexes, the relationships were found to be non-linear. In that case, the best fitting curves were obtained according to the exponential function described by the equation: *y* = *y*_0_ + A∙e^(*x*/*t*)^, where *y* denoted the percentage of inhibition and *x* denoted the complex concentration. The differences in the relationships (linear/non-linear) showed in Fig. 3 suggest a different mechanism of scavenging superoxide free radicals. However, the mechanism of the reaction of the complexes with O_2_^−^ is not easy to elucidate.

The antioxidant activities of the complexes towards O_2_^−^ were compared to each other. Based on the obtained relationships (Fig. 3), the complex concentrations causing the 50 % reduction of the concentration of the superoxide ion were calculated. The obtained values were referred to the concentration of ascorbic acid causing the same O_2_^−^ reduction effect measured at the same experimental conditions. In this way, ascorbic acid equivalents of the complexes were obtained. They are equal 0.35, 1.11 and 4.50 for VO(ODA), Ni(ODA) and Co(ODA), respectively. The results indicate that reactivity of VO(ODA) towards superoxide free radicals is higher than for the other two complexes and ascorbic acid used as a standard substance.

The conductometric measurements revealed that the stability of the complexes under study strongly depends on a solvent polarity. In the DMSO solution, the neutral oxydiacetate complexes behave as weak electrolytes and they do not exhibit the tendency to undergo the hydrolysis. Thus, the concentration of free metal ions in the system under study is expected to be very low, and the reactivity of complexes towards superoxide anions in the DMSO solution is mainly governed by the physicochemical properties of the oxovanadium(IV), cobalt(II) and nickel(II) ions bound to the ODA ligand. To explain the differences in the reactivity of the complexes towards superoxide ions, the ability of the coordination centres of the complexes for undergoing the reduction (M^ox^ + O_2_^−^ = M^red^ + O_2_) as well as the hard/soft-acid/base nature of the resulting species should be taken into account. The oxovanadium(IV) coordination centre has a rather low redox potential (V^IV^/V^III^: 0.337 V) and can be reduced to V^III^ [41]. Furthermore, the ODA ligand, containing hard oxygen donor atoms, has the greater ability to stabilize small hard vanadium(III) ions than the soft Ni(I) and Co(I) coordination centres. The reduction of the borderline ions is not favoured in the presence of oxygen donor ligands such as the oxydiacetate anions because they are not suitable for the stabilization of lower oxidation states (M^II^ + e = M^I^). For this reason, the resulting Ni(I) and Co(I) complexes are thermodynamically and kinetically unstable [42]. The above factors are responsible for lower reactivity of cobalt(II) and nickel(II) complexes than VO(ODA) towards O_2_^−^ under the experimental conditions.

### The Cytotoxicity of the Complexes

The dose and time-dependent effect of investigated compounds (in the concentration range of 1–500 μM) on a viability of the Human Dermal Fibroblasts adult (HDFa) cell line was tested at the mitochondrial level (MTT test) after 24 and 48 h of incubation. For nickel(II) and cobalt(II) complexes, no significant cytotoxic effects were observed after 24 h. At the highest concentration of the compounds (500 μM), the viability of the cells was no less than 95 %. An extension of the exposure time from 24 to 48 h triggers a slight decrease in the viability of the cells to about 90 and 75 % for the Co(II) and Ni(II) complex at 500 μM, respectively. It is interesting to note that only the oxovanadium(IV) complex exhibits the cytotoxic effect on fibroblast cells in the tested concentration range after 24 h of incubation and its cytotoxic activity increases in proportion to the exposure time. The cytotoxic properties of the complexes under study, expressed as lethal concentration (LC_25_, LC_50_ and LC_75_) values, are summarized in Table 5.

**Table 5:** The cytotoxic activity of VO(ODA), Co(ODA) and Ni(ODA) against the HDFa cell line expressed as the lethal concentration (LC) values (μM) after 24 h and 48 h of incubation (MTT test)

	NiODA	CoODA	VOODA
24-h exposure time			
LC_25_ [μM]	Immeasurable	Immeasurable	108.03
LC_50_ [μM]	Immeasurable	Immeasurable	295.74
LC_75_ [μM]	Immeasurable	Immeasurable	560.17
48-h exposure time			
LC_25_ [μM]	492.04	Immeasurable	27.03
LC_50_ [μM]	1309.71	Immeasurable	87.45
LC_75_ [μM]	2339.01	Immeasurable	180.53

LC values (μM) were estimated from dose–response curves using four-parameter logistic equations

### The Antiproliferative Activity of the Complexes

**Fig. 4:**
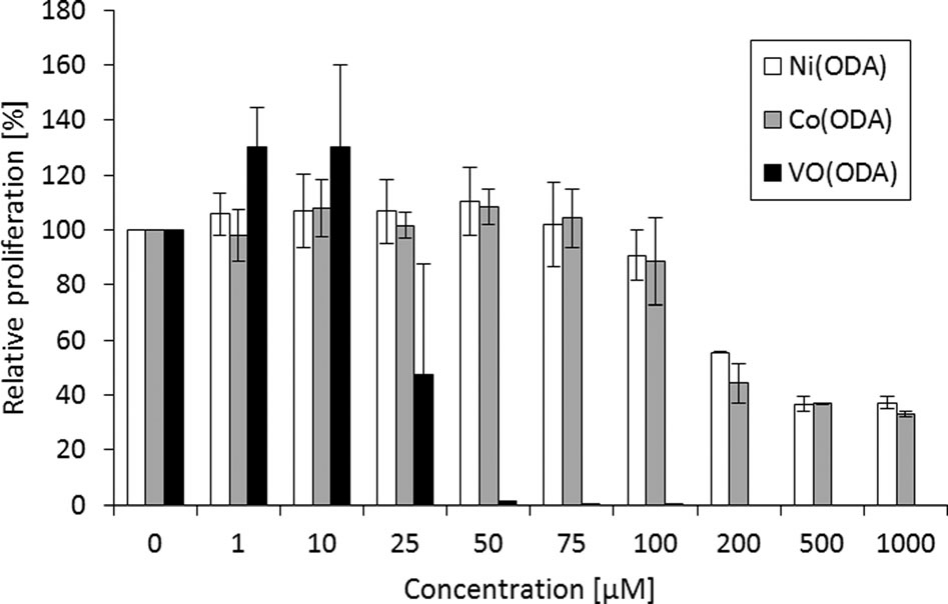
The influence of VO(ODA), Ni(ODA) and Co(ODA) on the relative proliferation of HDFa cells after 6 days of exposure to investigated complex compounds (based on 24-h BrdU incorporation by actively dividing cells). *Values* represent means of four different experiments (run in duplicate) ± SD

The dose-dependent (in the concentration range of 1–1000 μM) effect of the complexes on the HDFa cell proliferation was investigated by the measurement of BrdU (5-bromo-2′-deoxyuridine) incorporation by actively dividing cells after 6 days of culture in the presence of different concentrations of complexes (Fig. 4). Based on the obtained data, inhibitory concentrations (IC_25_, IC_50_ and IC_75_) for each compound were calculated and they are summarized in Table 6. The highest antiproliferative activity was found for oxovanadium(IV) complex (IC_50_ = 25 μM). At higher concentrations, the synergy effect, connected with the increase of the VO(ODA) cytotoxicity with the exposure time, on the antiproliferative activity was observed. The Co(II) and Ni(II) complexes exhibit the antiproliferative activity comparable to each other, and they cause the 50 % inhibition of cell proliferation at ca. 200 μM.

**Table 6:** The antiproliferative activity of the VO(ODA), Ni(ODA) and Co(ODA) complexes against HDFa cells after 6 days of exposure to investigated complex compounds (based on the 24-h BrdU incorporation by actively dividing cells)

6 day-exposure time			
	Ni(ODA)	Co(ODA)	VO(ODA)
IC_25_ (μM)	148.65	128.90	23.41
IC_50_ (μM)	219.97	175.08	24.86
IC_75_ (μM)	>1000	>1000	26.42

IC values (μM) were estimated from dose–response curves using four-parameter logistic equations

### The Cytoprotective Activity of the Complexes Against Oxidative Damage

To induce a cellular injury in the HDFa cell line and assess the cytoprotective activity of the complexes, the oxidative damage was generated exogenously by using hydrogen peroxide, H_2_O_2_. It is worth noticing that the activity of H_2_O_2_ as an oxidative damage-inducing factor was governed by the concentration of fetal bovine serum FBS in the cell culture medium, thus, 1 mM H_2_O_2_ in growth medium without FBS was used for the induction of oxidative damage resulting in the cellular death to about 80 % of cells after 1-h exposure.

**Fig. 5:**
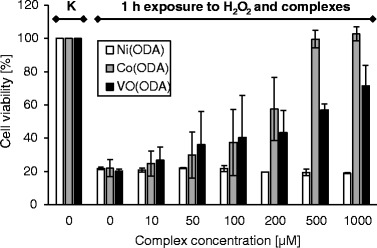
The viability of the HDFa cells after a 1-h incubation in the presence of 1 mM H_2_O_2_ and different concentrations of investigated complex compounds. *K* denotes cells not treated with H_2_O_2_ nor complexes (control cells). *Values* represent means of three different experiments (run in triplicate) ± SD

**Fig. 6:**
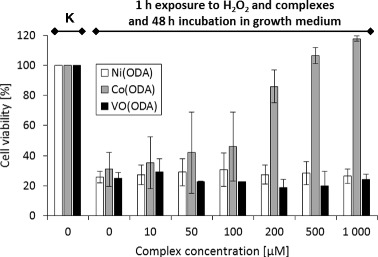
The viability of HDFa cells after an additional 48-h incubation in the growth medium following a 1-h incubation in the presence of 1 mM H_2_O_2_ and different concentrations of investigated complex compounds. *K* denotes cells not treated with H_2_O_2_ nor complexes (control cells). *Values* represent means of three different experiments (run in triplicate) ± SD

Screening of cytoprotective properties of VO(IV), Ni(II) and Co(II) complexes has shown that Co(ODA) exhibits the highest dose- and time-dependent protective effect against the oxidative damage. The results of the MTT test (Figs. 5 and 6) revealed that the viability of human fibroblasts treated with 1 mM H_2_O_2_ together with the complex for 1 h increases in proportion to the amount of Co(ODA) in the system (Fig. 5). The viability of cells increases up to ca. 100 % in the case of treatment with 500 μM Co(ODA) and is comparable to the untreated cells (Fig. 5). The ability of cells, first exposed to H_2_O_2_ and Co(ODA), to grow and proliferate at similar levels to untreated control cells after an additional 48-h incubation in the normal growth medium, confirms the cytoprotective activity of this complex against the oxidative damage (Fig. 6). Interestingly, also VO(ODA), to some extent, exhibits the cytoprotective activity against the oxidative damage to cells at the short, 1-h exposure time (Fig. 5), but due to its strong cytotoxic activity, VO(ODA) itself induces the cell death and the effect is visible after the additional 48-h incubation of cells in the growth medium (Fig. 6). A different situation is seen for the Ni(ODA) complex. It has no influence on the viability of cells, regardless of the dose used together with H_2_O_2_ (Figs. 5 and 6).

### The Antimicrobial Activity of the Complexes

The antimicrobial activity of the complexes studied was evaluated against following bacteria species: *Bacillus subtilis*, *Escherichia coli*, *Enterococcus faecalis*, *Pseudomonas aeruginosa*, *Staphylococcus aureus* and *Staphylococcus epidermidis*. The minimum inhibitory concentrations (MICs) were determined for each complex based on the change of the optical density of bacterial cultures grown over the complex concentration range 1–2048 μg/mL (Table 7). The study revealed that all complexes inhibit the growth of bacteria under study, but their activity depends on the complex concentration. The highest antimicrobial activity was found for the Co(ODA) complex.

**Table 7:** MIC and MBC values (μg/mL) for the complexes obtained in the broth dilution test

Bacteria	Ni(ODA) (μg/mL)		Co(ODA) (μg/mL)		VO(ODA) (μg/mL)	
	MIC	MBC	MIC	MBC	MIC	MBC
*B. subtilis*	512	–	256	–	2048	–
*E. coli*	1024	–	512	1024	2048	–
*E. faecalis*	1024	–	256	–	1024	1024
*P. aeruginosa*	1024	–	256	–	1024	–
*S. aureus*	1024	–	256	512	1024	2048
*S. epidermidis*	512	2048	256	2048	2048	2048

To assess whether the antimicrobial activity of complexes was bacteriostatic or bactericidal, for each culture medium, where no visible growth was observed, the number of bacteria colonies formed on LB plates was counted after an overnight incubation at 310.15 K. MBC values were estimated and the results are summarized in Table 7. The complexes exhibit the bacteriostatic activity against most of bacterial strains. Furthermore, the bactericidal activity against *S. epidermidis* has been found for Ni(ODA). The Co(ODA) complex shows the bactericidal activity against *E. coli*, *S. aureus* and *S. epidermidis* whereas VO(ODA) against *E. faecalis*, *S. aureus* and *S. epidermidis*.

Zones of inhibition of bacterial growth for investigated complexes are listed in Table 8. The most effective values were obtained for Co(ODA) at the amount of 1000 μg per disc, and the activity of this complex was similar to that of 100 μg of neomycin against all tested bacteria strains except *B. subtilis*. Ni(ODA) showed some activity against *B. subtilis* and *S. epidermidis* and VO(ODA) against *B. subtilis*, *E. faecalis* and *S. epidermidis*, but in both cases, zones of inhibition were lower comparing to Co(ODA).

**Table 8:** Zones of complete bacterial growth inhibition (mm) for complexes obtained in the disc diffusion test

Bacteria	Zone of growth inhibition (mm)									
	Ni(ODA)			Co(ODA)			VO(ODA)			Neo
	(μg/disc)									
	100	500	1000	100	500	1000	100	500	1000	100
*B. subtilis*	–	8	8	–	9	11	–	–	7	21
*E. coli*	–	–	–	–	12	14	–	–	–	15
*E. faecalis*	–	–	–	–	10	15	–	9	13	16
*P. aeruginosa*	–	–	–	–	14	18	–	–	–	17
*S. aureus*	–	7	8	7	12	17	–	–	–	18
*S. epidermidis*	–	–	–	8	19	23	–	–	12	25

Neomycin (Neo) was used as a control

## Conclusions

The potentiometric and conductometric titration methods have successfully been applied to characterize the stability of the oxydiacetate complexes of oxovanadium(IV), cobalt(II) and nickel(II) in the dimethylsulfoxide (DMSO)-water (H_2_O) binary mixtures.

The stability constant values for the complexes studied increase in the sequence Co(ODA) ≈ Ni(ODA) < VO(ODA). This finding is in a good agreement with the classification of the donor—acceptor atoms based on the HSAB theory. A softer character of Co(II) and Ni(II) with respect to VO(IV) results in the formation of less stable complexes with the ligands containing hard donor atoms. On the other hand, the cobalt(II) and nickel(II) complexes, in contrary to VO(ODA), are stable in a much broader pH range. Furthermore, conductometric measurements reveal that the stability of the complexes increases in proportion to the amount of DMSO in the system. Thus, in media with low dielectric constants, the complexes behave as weak electrolytes.

Based on the NBT assay, it has been found that all the complexes studied are capable to scavenge the superoxide free radical. Their reactivity towards O_2_^−^ is governed by the ability of the ligand to stabilize the lower oxidation states of the coordination centre. For this reason, VO(ODA) has been found to show the highest superoxide dismutase (SOD) activity.

The cytoprotective activity of the complexes against the oxidative damage of fibroblast cells generated by hydrogen peroxide strongly depends on the kind of the coordination centre of the complex. The cobalt(II) complex has been found to show the highest cytoprotective activity in contrast to the nickel(II) complex that does not exhibit cytoprotective activity, regardless of the dose used. Unexpectedly, VO(ODA) has been found to exhibit the cytotoxic activity on fibroblast cells. However, the oxovanadium(IV) complex exhibits the cytoprotective activity against the oxidative damage to cells at a short time, but due to its cytotoxic effect itself induces the cell death after further exposure.

The oxovanadium(IV), cobalt(II) and nickel(II) complexes studied inhibit the growth of bacteria under study. The highest antimicrobial activity is revealed by the Co(ODA) complex.
